# The *Clinical Proteomics *editorial team would like to announce a change in the volume listings for the journal

**DOI:** 10.1186/1559-0275-7-1

**Published:** 2011-12-02

**Authors:** Clinical Proteomics

**Affiliations:** 1 c/o BioMed Central, 236 Gray's Inn Road, London, WC1X 8HB, UK

## Publisher's note

Owing to the technical process involved in transferring the journal to its new publisher, BioMed Central, Volume 7 of *Clinical Proteomics *will now contain only this publisher's note. Volume 8 will contain all articles published in 2010, and all other articles published throughout 2011.

As an online only journal *Clinical Proteomics *will, from this point forwards, publish one volume annually, with Volume 9 beginning January 2012.

Any questions can be directed to editorial@clinicalproteomicsjournal.com.

